# Risk factors for diastasis recti abdominis and its correlation with pelvic organ prolapse among postpartum women in southwest China: a retrospective case-control study

**DOI:** 10.3389/fgwh.2025.1693283

**Published:** 2026-01-22

**Authors:** Dehua Wan, Ling Guo, Shuwen Cheng, Ling Ren, Taizhou Qin, Xueping Zhang, Huarong Wang, Zhongyan Zheng, Xiaoqin Gan, Tianjiao Liu, Yonghong Lin

**Affiliations:** 1 Guidance Center of Women’s Healthcare, Chengdu Women’s and Children’s Central Hospital, School of Medicine, University of Electronic Science and Technology of China, Chengdu, China; 2 Department of Chronic and Non-communicable Disease Control and Prevention, Sichuan Center for Disease Control and Prevention, Chengdu, China; 3 Department of Gynecology, Chengdu Women’s and Children’s Central Hospital, School of Medicine, University of Electronic Science and Technology of China, Chengdu, China

**Keywords:** diastasis recti abdominis (DRA), pelvic organ prolapse (POP), postpartum healthcare, pelvic floor dysfunction, risk factors analysis

## Abstract

**Background:**

Diastasis recti abdominis (DRA) is a prevalent postpartum condition characterized by separation of the rectus abdominis muscles. It has been linked to lumbopelvic pain, pelvic floor dysfunction, and urinary incontinence. However, large-scale epidemiological data from Chinese populations and study investigating its relationship with pelvic organ prolapse (POP) are limited.

**Methods:**

This is a retrospective case-control study which analyzed 4,426 women at 6th week postpartum at Chengdu Women's and Children's Central Hospital between January 2019 and January 2025. DRA was diagnosed by ultrasonography, and POP was staged using the POP-Q system. Maternal, obstetric, and pelvic floor variables were collected. Independent risk factors of DRA were identified through ordinal logistic regression analyses. Spearman correlation was used to examine the relationship between DRA severity and POP stage. A two-tailed *p* value less than 0.05 is regarded as statistically significant.

**Results:**

DRA was detected in 65.6% of participants, with 52.6% classified as mild to moderate. High neonatal birth weight (≥3.5 kg) is independent risk factor for DRA (OR 2.43, 95% CI 1.75–3.35). Multiparas were more than twice as likely to develop DRA compared to their nulliparous counterparts (OR 2.67, 95% CI 1.30–5.45). Vaginal delivery (OR 0.45, 95% CI 0.40–0.51) and type I pelvic floor muscle strength grade II to IV were associated with lower risks for DRA. More than half of women in each age group (20–29, 30–39, and ≥40-year-old) presented with both DRA and POP. Spearman analysis showed a significant negative correlation between DRA severity and POP stage (*ρ* = –0.220, *p* < 0.001).

**Conclusion:**

Diastasis recti abdominis is highly prevalent among Chinese postpartum women and is influenced by parity, birth weight, gestational weight gain, and maternal BMI. Vaginal delivery and moderate (Grade II–IV) strength of Type I pelvic floor muscles were found to be protective against Diastasis Recti Abdominis.

## 1 Background

Diastasis recti abdominis (DRA) refers to the separation of the rectus abdominis muscles along the linea alba and is a common condition observed during pregnancy and the postpartum period (1). Even though there is no specified symptoms for definitively diagnosing DRA, patients often experience lumbopelvic pain, and about 60% have concurrent pelvic floor dysfunction (2). Moreover, the incidence of postpartum urinary incontinence is significantly higher among DRA patients (3, 4). The reported prevalence of DRA varies considerably depending on the evaluation methods, cut-off values, timing of assessment, and study population. Many studies reported prevalence of approximately 60% at 6 weeks postpartum (5). A recent investigation among adult Chinese women also revealed a high DRA prevalence of 28.4% (6). Significantly high prevalence of DRA and its impact on lumbopelvic and pelvic floor health demand for targeted assessment that shall guide workable prevention and treatment measures.

While physiological and mechanical changes during pregnancy, as well as factors such as mode of delivery, are believed to contribute to the development of DRA among postpartum women, its specific risk factors remain inadequately defined. Variables including maternal age, body mass index (BMI), delivery method, neonatal birth weight, and physical activity have all been implicated, yet current findings remain inconsistent across studies. Moreover, studies analyzing the association between DRA and pelvic organ prolapse (POP) remain limited. A large population of Chinese postpartum women suffer from DRA and POP; however, most of the available evidence originates from Western populations, and large-scale epidemiological studies among Chinese women remain comparatively limited.

To bridge this knowledge gap, the present study aimed to examine the prevalence of DRA and its associated obstetric and demographic factors in a large population of postpartum women from Chengdu, Sichuan Province, China. Additionally, we aim to analyze the association between DRA and parameters related to POP using this large-sample dataset. This study aims to generate evidence-based insights into the risk factors for DRA to inform prenatal counselling and pregnancy management strategies for its prevention. The findings are also intended to guide postnatal care, particularly rehabilitation approaches in a specified context of maternal healthcare.

## 2 Method

### 2.1 Study design and setting

**Figure 1:**
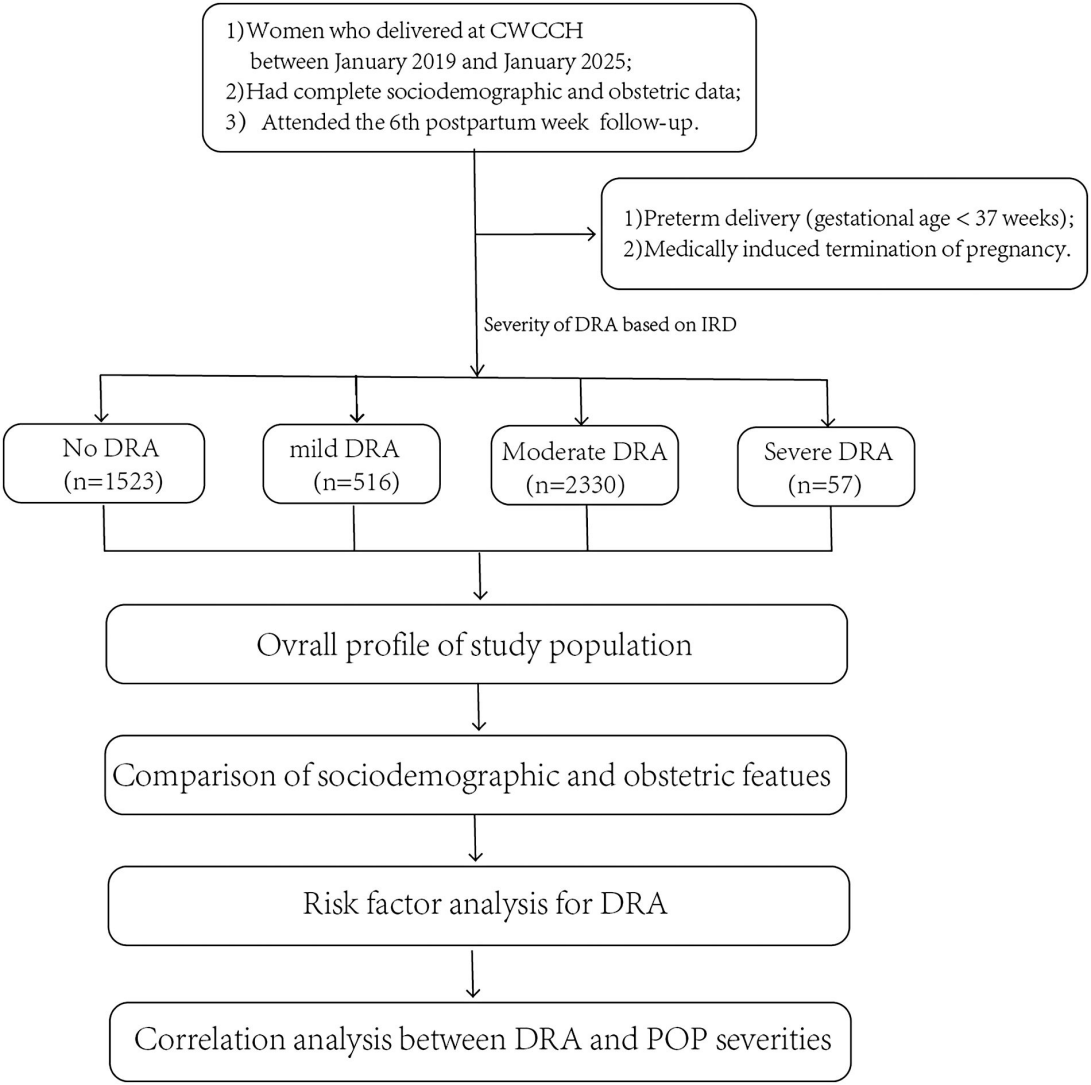
Flowchart of case selection, grouping, and analytical procedures. CWCCH, Chengdu Women's and Children's Central Hospital; DRA, diastasis recti abdominis; IRD, inter-rectus distance; POP, pelvic organ prolapse.

This retrospective case–control study investigated the occurrence of DRA at 42 days postpartum among women who delivered at Chengdu Women's and Children's Central Hospital (CWCCH) between January 2019 and January 2025 (Figure 1). CWCCH is a tertiary university-affiliated hospital under the University of Electronic Science and Technology of China, specializing in maternal and child health care. It serves as a regional referral and clinical treatment center for maternal and child health care in Southwest China, with an annual delivery volume of approximately 15,000 to 20,000 births and a catchment population of around 20 million, primarily from Chengdu and its surrounding prefectures and counties (7).

### 2.2 Subjects

Participants were retrospectively selected based on the following criteria:

Inclusion criteria: 1) Delivered at CWCCH during the study period; 2) Had complete sociodemographic and obstetric data; 3) Attended the 6th postpartum week follow-up.

Exclusion criteria: 1) Preterm delivery (gestational age < 37 weeks); 2) Medically induced termination of pregnancy.

### 2.3 Definition of terms

DRA: DRA is the separation of the rectus abdominis muscles along the linea alba. This study adopted the ultrasound diagnostic and classification criteria for DRA proposed in the domestic *Expert Consensus on the Diagnosis and Treatment of Postpartum Diastasis Recti Abdominis (2023)* and the criteria applied in previous studies (8–14). Based on the guidelines of the European Hernia Society, patients were classified into four groups according to the value of inter-rectus distance (IRD): no DRA, mild DRA (IRD less than 2.9 cm), moderate DRA (IRD 3–4.9 cm), and severe DRA (IRD >5 cm) (10, 15).

POP: POP refers to the descent of pelvic organs into or beyond the vaginal canal. The diagnosis and staging of POP in present study were conducted in accordance with *the ACOG Practice Bulletin No. 214, the 2020 Chinese Guidelines for the Diagnosis and Treatment of POP,* and *the Pelvic Organ Prolapse Quantification (POP-Q) system* developed by the International Continence Society (ICS) and the International Urogynecology Association (IUGA) (16–21). POP was primarily identified based on patient-reported symptoms and clinical examination, with additional tests performed when necessary (Table 1).

**Table 1:** Staging criteria for pelvic organ prolapse.

Stage	Definition
0	No detectable prolapse; all reference points are at −3 cm, and point C or D lies between –TVL and –(TVL–2) cm.
I	Minor descent; the leading edge of the prolapse remains >1 cm above the hymen.
II	The most dependent point is within 1 cm above or below the hymenal plane.
III	Prolapse extends more than 1 cm beyond the hymen but remains above TVL–2 cm.
IV	Complete vaginal eversion; the protrusion reaches or exceeds (TVL–2) cm, indicating total prolapse.

Pelvic floor dysfunction (PFD): PFD is defined as a reduction in strength affecting either type I or type II muscle fibers. Type I pelvic muscle fibers primarily function to maintain the normal position of pelvic organs, whereas type II pelvic fibers play a key role in urinary and fecal continence as well as sexual function.

### 2.4 Study procedures

The study workflow comprised three key steps: case selection, data collection, and statistical analysis (Figure 1). We screened eligible cases from the HIS of CWCCH hospital information system according to the aforementioned inclusion and exclusion criteria. Demographic data and follow-up records from the 42nd postpartum day, including assessments of DRA, POP, and pelvic floor muscle strength, the diagnostic methods and grading criteria for which are described in detail below. Data collection included sociodemographic and perinatal variables such as maternal age, occupational physical activity level (mainly sedentary or physically active), pre-pregnancy body mass index (BMI, kg/m^2^), neonatal birth weight (kg), gestational weight gain (kg), gravidity, parity, mode of delivery, and the use of forceps during birth. At the 6th postpartum week, DRA and POP were diagnosed and classified during follow-up visits, accompanied by pelvic floor muscle strength assessment and other postpartum examinations. Subsequently, the overall demographic characteristics of the participants were analyzed. Based on the presence and severity of DRA, participants were categorized into four groups: no DRA, mild DRA, moderate DRA, and severe DRA. Sociodemographic and obstetric characteristics, pelvic floor muscle strength, and POP grading were compared across these groups. Further analyses on DRA risk factors and the distribution of POP and DRA comorbidity across different age groups, and the correlation between DRA and POP severities were also performed.

## 3 DRA, POP and pelvic muscle strength assessment procedure

### 3.1 Diagnosis and classification procedure for DRA

Ultrasonographic examinations were performed using a handheld wireless color Doppler ultrasound system (PR363P, PUBO Medical Equipment, Shenzhen, China) with a probe frequency of 7.5 MHz during the routine six-week postpartum follow-up. All patients were examined in the supine resting position. High-frequency transverse ultrasound images were used to measure the IRD at the medial edges of the rectus abdominis muscle bellies. When the muscle boundaries were not clearly visualized, tissue harmonic imaging was applied. For cases where the separation exceeded 4 cm, panoramic ultrasound imaging was employed to extend the field of view; for separations greater than 8 cm, compound imaging through left-right stitching was used to generate a continuous image.

### 3.2 POP classification and grading procedure

Assessment of POP included anatomical localization and severity grading of prolapse, along with evaluation of related urinary symptoms such as stress incontinence or overactive bladder. Classification was determined by the furthest descent of the prolapsed organ relative to the hymenal ring, considering one or more pelvic compartments (22, 23). The POP-Q staging system is defined as follows:

### 3.3 Clinical assessment of pelvic muscle strength

The pelvic muscle strength was graded on a six-point scale ranging from 0 to V, where Grade V denoted the best strength and Grade 0 indicated complete absence of contraction; thus, lower grades reflected poorer muscle function. A classification of Grade III or below was considered indicative of dysfunction, whereas Grades IV and V were regarded as normal function (24, 25). The evaluation of type I fiber strength was based on the endurance of voluntary vaginal contraction sustained at ≥40% of the individual's maximal contraction intensity. Endurance capacity was divided into six categories (Grade 0–V), corresponding to the ability to maintain contraction for 0, 1, 2, 3, 4, and ≥5 s, respectively. Type II fiber strength was determined according to the number of rapid voluntary vaginal contractions that could reach at least 60% of maximal contraction intensity within a 15-second testing period. The number of measurable contraction peaks was recorded from 0 to ≥5 and used to define Grades 0 through V, respectively.

### 3.4 Statistical analysis

Data were saved in the form of Microsoft Excel (Microsoft Corp., Redmond, WA, USA) file and statistically analyzed using SPSS version 27.0 (IBM Corp., Armonk, NY, USA). Categorical variables were expressed as counts and percentages, and compared via the Mann–Whitney U test. Variables found to be statistically significant in univariate analyses or potentially correlates with DRA in the literatures were subsequently included in an ordinal logistic regression analysis to determine predictors of DRA (1, 5, 10, 26–28). The correlation between DRA and POP staging was further evaluated using Spearman's rank correlation test. Statistical significance was defined as a two-sided *p*-value of less than 0.05.

## 4 Results

### 4.1 Sociodemographic characteristics and DRA profile of the studied group

Out of 4,426 postpartum women, the majority of participants were aged between 30 and 39 years (51.8%), followed by those aged 20–29 years (46.5%), while only a small proportion (1.7%) were aged ≥40 (Table 2). Majority of women (85.7%) were engaged primarily in sedentary work, with the remainder involved in physical active work. Most participants fell within the normal BMI range (18.5–23.9 kg/m^2^, 66.7%), with underweight (<18.5 kg/m^2^) and obesity (≥28.0 kg/m^2^) accounting for 4.20% and 4.0%, respectively. Notably, more than half of the participants (52.6%) exhibited mild to moderate diastasis recti (IRD 3–4.9 cm), while 34.4% had no DRA, and only 1.3% demonstrated severe separation (IRD ≥5 cm).

**Table 2:** Overall sociodemographic profile and distribution of DRA grades.

Characteristic		*N*	Proportion (%)
Number of participants		4,426	100
Age group (years)	20–29	2,059	46.5
	30–39	2,292	51.8
	≥40	75	1.7
Occupational physical activity level	Mainly sedentary	3,791	85.7
	Mainly physically active	635	14.4
BMI (kg/m^2^)	< 18.5	186	4.2
	18.5–23.9	2,952	66.7
	24.0–27.9	1,112	25.1
	≥28.0	176	4.0
DRA grade^a^	None	1,523	34.4
	Mild	516	11.7
	Moderate	2,330	52.6
	Severe	57	1.3

BMI, body mass index; DRA, Diastasis recti abdominis; IRD, inter-rectus distance.

a DRA are classified into four groups according to the value of inter-rectus distance (IRD): no DRA, mild DRA (IRD 0–2.9 cm), moderate DRA (IRD 3–4.9 cm), and severe DRA (IRD >5 cm).

### 4.2 The association of demographic and clinical characteristics to severity of DRA in the studies group

The distribution of DRA severity varied significantly across several demographic and clinical characteristics (Table 3). Women aged 30–39 years and ≥40 years had a higher proportion of moderate-to-severe DRA compared with those aged 20–29 years (*P* < 0.001). In contrast, BMI demonstrated a clear gradient: women with higher BMI categories (≥24.0 kg/m^2^) were more likely to have severe DRA than those with normal BMI (*P* < 0.001). The prevalence of severe DRA increasing from 0.7% in infants weighing 2.5–2.9 kg to 2.5% in those ≥4.0 kg (*P* < 0.001). Excessive gestational weight gain (≥15 kg) was associated with a 1.2-fold higher risk of developing DRA compared with women who gained less than 15 kg during pregnancy (*P* = 0.002). Higher gravidity and parity were associated with greater severity of DRA, particularly among women with parity ≥3, in whom 73.7% had a separation of ≥3 cm (*P* < 0.001). Forceps delivery was related to a higher proportion of moderate-to-severe DRA (*P* < 0.001). Finally, pelvic floor muscle strength was strongly correlated with DRA severity: lower grades of both type I and type II muscle strength were associated with wider separations, whereas women with higher muscle grades (IV–V) had a lower prevalence of severe DRA (*P* < 0.001). Occupational physical activity level (mainly sedentary vs. physically active), POP stage, and delivery mode showed no significant differences in the distribution of DRA severity (*p* > 0.05).

**Table 3:** Distribution of DRA severities across different demographic and clinical features.

Characteristic	DRA severity^a^	None *n* (%)	Mild *n* (%)	Moderate *n* (%)	Severe *n* (%)	*P* value
	Categories					
Age group (years)	20–29	738 (35.8)	289 (14.0)	1,020 (49.5)	12 (0.6)	<0.001
	30–39	761 (33.2)	223 (9.7)	1,264 (55.2)	44 (1.9)	
	≥40	24 (32.0)	4 (5.3)	46 (61.3)	1 (1.3)	
Occupational physical activity level	Mainly sedentary	1,308 (34.5)	444 (11.7)	1,986 (52.4)	53 (1.4)	0.810
	Mainly physically active	215 (33.9)	72 (11.3)	344 (54.2)	4 (0.6)	
BMI (kg/m^2^)	< 18.5	73 (39.3)	27 (14.5)	86 (46.2)	0	<0.001
	18.5–23.9	1,042 (35.3)	372 (12.6)	1,501 (50.9)	37 (1.3)	
	24.0–27.9	356 (32.0)	97 (8.7)	642 (57.7)	17 (1.5)	
	≥28.0	52 (29.6)	20 (11.4)	101 (57.4)	3 (1.7)	
Birth weight (kg)	2.5–2.9	404 (39.0)	144 (14.0)	481 (46.4)	7 (0.7)	<0.001
	3.0–3.4	766 (36.3)	253 (12.0)	1,073 (50.8)	20 (1.0)	
	3.5–3.9	311 (28.9)	104 (9.7)	637 (59.2)	25 (2.3)	
	≥4.0	42 (20.9)	15 (7.5)	139 (69.2)	5 (2.5)	
Gestational weight gain (kg)	<15	1,159 (35.6)	385 (11.8)	1,672 (51.4)	39 (1.2)	0.002
	≥15	364 (31.1)	131 (11.2)	658 (56.2)	18 (1.5)	
Gravidity	1	799 (36.9)	293 (13.6)	1,055 (48.8)	16 (0.7)	<0.001
	2	400 (32.6)	127 (10.4)	675 (55.1)	24 (2.0)	
	≥3	324 (31.2)	96 (9.3)	600 (57.9)	17 (1.6)	
Parity	1	1,184 (36.9)	403 (12.6)	1,592 (49.6)	31 (1.0)	<0.001
	2	333 (28.3)	109 (9.3)	710 (60.3)	26 (2.2)	
	≥3	6 (15.8)	4 (10.5)	28 (73.7)	0 (0.00)	
Delivery mode	Cesarean section	570 (26.3)	202 (9.3)	1,352 (62.4)	42 (1.9)	0.084
	Vaginal delivery	953 (42.2)	314 (13.9)	978 (43.3)	15 (0.6)	
Forceps delivery	No	1,431 (34.2)	481 (11.5)	2,217 (53.0)	54 (1.3)	<0.001
	Yes	92 (37.9)	35 (14.4)	113 (46.5)	3 (1.2)	
Type I pelvic floor muscle strength	0	75 (28.0)	30 (11.2)	161 (60.1)	2 (0.8)	<0.001
	I	822 (31.1)	293 (11.1)	1,500 (56.8)	28 (1.1)	
	II	338 (38.6)	106 (12.1)	413 (47.2)	19 (2.2)	
	III	211 (42.7)	68 (13.8)	208 (42.1)	7 (1.4)	
	IV	64 (54.7)	17 (14.5)	36 (30.8)	0	
	V	13 (46.4)	2 (7.1)	12 (42.9)	1 (3.6)	
Type II pelvic floor muscle strength	0	58 (29.7)	28 (14.4)	107 (54.9)	2 (1.0)	<0.001
	I	587 (29.7)	219 (11.1)	1,149 (58.1)	22 (1.1)	
	II	378 (36.5)	131 (12.6)	512 (49.4)	15 (1.5)	
	III	328 (38.5)	97 (11.4)	412 (48.4)	15 (1.8)	
	IV	149 (46.1)	38 (11.8)	133 (41.2)	3 (0.9)	
	V	23 (53.5)	3 (7.0)	17 (39.5)	2 (1.0)	
POP grade	Without POP	359 (32.1)	131 (11.7)	613 (54.8)	15 (1.3)	0.345
	POP I	1,003 (35.3)	341 (12.0)	1,464 (51.5)	37 (1.3)	
	POP II	161 (34.8)	44 (9.5)	253 (54.6)	5 (1.1)	

Mann–Whitney U test.

BMI, body mass index; DRA, diastasis recti abdominis; POP, Pelvic Organ Prolapse.

a DRA are classified into four groups according to the value of inter-rectus distance (IRD): no DRA, mild DRA (IRD 0–2.9 cm), moderate DRA (IRD 3–4.9 cm), and severe DRA (IRD >5 cm).

### 4.3 Background risk factors and their association to the severity of DRA

Higher fetal birth weight emerged as a robust predictor of DRA (Table 4). Compared with infants weighing 2.5–3.0 kg, those in the 3.0–3.5 kg group had a modestly elevated risk of DRA, with an OR of 1.18 (95% CI: 1.02–1.36, *P* = 0.031). The risk was further increased among infants weighing 3.5–4.0 kg (OR: 1.67, 95% CI: 1.40–1.98, *P* < 0.001). Notably, infants with a birth weight ≥4.0 kg demonstrated the highest likelihood, with a 2.43-fold increase (OR: 2.43, 95% CI: 1.75–3.35, *P* < 0.001). Likewise, greater parity was significantly linked to DRA, with women who had three or more deliveries facing a 2.67-fold higher likelihood (OR: 2.67, 95% CI: 1.30–5.45, *P* = 0.007) compared to primiparous women. Vaginal delivery was associated with a significantly reduced likelihood of DRA (OR: 0.45, 95% CI: 0.40–0.51, *P* < 0.001). Furthermore, increased type I muscle strength grades II to IV were also found relevant to lower risks of DRA, with progressively lower odds ratios at higher muscle grades. Specifically, grade II was associated with an OR of 0.60 (95% CI: 0.38–0.93, *P* = 0.023), grade III with an OR of 0.49 (95% CI: 0.30–0.78, *P* = 0.003), and grade IV with an OR of 0.34 (95% CI: 0.19–0.62, *P* < 0.001).

**Table 4:** DRA risk factor analysis on sociodemographic and obstetric characteristics.

Characteristic	Categories	*B* coefficient	Odds ratio (95% CI)	*P* value
Age group (years)	20–29^a^	–	–	–
	30–39	0.017	1.02 (0.89–1.16)	0.798
	≥40	0.077	1.08 (0.67–1.74)	0.753
Occupational physical activity level	Mainly sedentary^a^	–	–	–
	Mainly physically active	–0.06	0.94 (0.79–1.12)	0.490
BMI (kg/m^2^)	<18.5^a^	–	–	–
	18.5–23.9	0.131	1.14 (0.85–1.53)	0.380
	24.0–27.9	0.187	1.21 (0.88–1.65)	0.236
	≥28.0	0.186	1.20 (0.80–1.82)	0.380
Birth weight (kg)	2.5–2.9^a^	–	–	–
	3.0–3.4	0.162	1.18 (1.02–1.36)	0.031
	3.5–4.0	0.512	1.67 (1.40–1.98)	<0.001
	≥4.0	0.886	2.43 (1.75–3.35)	<0.001
Gestational weight gain (kg)	<15^a^	–	–	–
	≥15	0.183	1.20 (1.05–1.37)	0.008
Gravity	1^a^	0^a^		
	2	0.04	1.04 (0.89, 1.22)	0.616
	≥3	−0.123	0.88 (0.73, 1.07)	0.209
Parity	1^a^	–	–	–
	2	0.475	1.61 (1.35–1.92)	<0.001
	≥3	0.981	2.67 (1.30–5.45)	0.007
Delivery mode	Cesarean^a^	–	–	–
	Vaginal	–0.804	0.45 (0.40–0.51)	<0.001
Type I pelvic floor muscle strength	0^a^			
	I	−0.295	0.74 (0.49, 1.12)	0.160
	II	−0.516	0.60 (0.38, 0.93)	0.023
	III	−0.721	0.49 (0.3, 0.78)	0.003
	IV	−1.073	0.34 (0.19, 0.62)	<0.001
	V	−0.56	0.57 (0.22, 1.48)	0.250
Type II pelvic floor muscle strength	0^a^			
	I	0.320	1.38 (0.86, 2.2)	0.179
	II	0.067	1.07 (0.65, 1.75)	0.791
	III	0.092	1.1 (0.66, 1.82)	0.722
	IV	−0.098	0.91 (0.52, 1.58)	0.729
	V	−0.334	0.72 (0.3, 1.69)	0.445

Ordinal logistic regression analysis.

BMI, body mass index.

a As reference group.

### 4.4 Prevalence of POP and its correlation with DRA staging

Among women aged 20–29 years, 30.3% had POP, 18.5% had DRA, and 51.2% had both (Table 5). In the 30–39 years group, the prevalence was 27.1%, 18.85%, and 54.0%, respectively. The distribution of patients with POP, DRA, and concurrent DRA and POP did not differ significantly across age groups (*p* = 0.263). For women aged ≥40 years, 28.2% had POP, 16.9% had DRA, and 54.9% had both. The spearman's rank correlation analysis in Table 6 suggested that higher vertical degree was associated with lower separation degree (*ρ* = −0.220, *p* < 0.001).

**Table 5:** Distribution of POP and DRA comorbidity in different age groups.

Age group (years)	POP *n* (%)	DRA *n* (%)	POP + DRA *n* (%)	*p*-value
20–29	574 (30.3)	351 (18.5)	970 (51.2)	0.263
30–39	570 (27.1)	396 (18.9)	1,135 (54.0)	
≥40	20 (28.2)	12 (16.9)	39 (54.9)	

Chi square test.

POP, Pelvic Organ Prolapse; DRA, Diastasis Recti Abdominis.

**Table 6:** Correlation analysis between DRA stages and POP grades.

Variable	*ρ* (Spearman's rho)	*p*-value
POP stages vs. DRA grades	−0.220	<0.001

Spearman's rank correlation test.

DRA, Diastasis Recti Abdominis; POP, Pelvic Organ Prolapse.

## 5 Discussion

This study revealed that DRA is highly prevalent, with over half of the participants exhibiting moderate separation of the rectus abdominis muscles [defined as (IRD) 3–5 cm]. Several maternal and neonatal factors, including age, BMI, neonatal birth weight, gestational weight gain, gravidity, parity, and type I pelvic floor muscle strength grade II to IV, were significantly associated with the occurrence of DRA (28).

The prevalence of DRA in our cohort at sixth week postpartum ranged from 60% to 70%, confirming that this condition is highly common in the early postpartum period (2, 3). A Norwegian longitudinal study similarly reported that the prevalence of DRA increased from 33.1% at 21 weeks of gestation to approximately 60.0% at 6 weeks postpartum, before gradually declining to 45.4% at 6 months and 32.6% at 12 months postpartum (5). These findings suggest that DRA may partially resolve over time in some women, while others may experience persistent or worsening symptoms, possibly due to individual differences in tissue healing, physical activity, or preexisting musculoskeletal conditions.

The high prevalence of DRA among perinatal and postpartum women is underpinned by distinct physiological and anatomical characteristics (3, 29). The anatomical basis of DRA lies in the separation of the rectus abdominis muscles, primarily caused by progressive stretching and thinning of the linea alba, a midline fibrous structure predominantly composed of collagen (1). As the uterus expands during pregnancy, increased intra-abdominal pressure exerts mechanical tension on the abdominal wall, which leads to elongation and decreased tensile strength of the linea alba (30). At the same time, elevated levels of pregnancy-related hormones such as relaxin, estrogen, and progesterone promote softening of connective tissues, further weakening fascial integrity and facilitating muscle displacement (9–13).

We found that higher neonatal birth weight (≥3.5 kg) and excessive gestational weight gain were significantly associated with severer DRA. These factors contribute to increased intra-abdominal pressure and greater distension of the abdominal wall during pregnancy, thereby exacerbating fascial stretching and muscle separation. Similarly, women with multiple previous deliveries (multiparity) and multiple gestations (e.g., twins or higher-order births) are exposed to repeated or compounded abdominal wall stress, which may overwhelm the tissue's ability to recover between pregnancies. Additionally, higher weight gain during pregnancy was also correlated with the occurrence of DRA. These findings are consistent with prior studies highlighting parity and fetal macrosomia as important predictors for developing DRA (3, 5, 6, 10, 26, 28).

The impact of delivery mode on the development of DRA remains controversial. Some studies have suggested that cesarean section may lower the risk of DRA or no specific impact of delivery mode on the occurrence of DRA (31). Theoretically, when cesarean section was performed, as the abdominal wall undergoes less strain from pushing during labor, it would thereby reducing mechanical tension on the linea alba. Conversely, other reports argue that vaginal delivery may serve as a protective factor for developing DRA (26). In our study, forceps-assisted vaginal deliveries were identified as a significant risk factor for DRA. This association may be explained by the prolonged second-stage labor, elevated intra-abdominal pressure, and greater mechanical stress on the abdominal wall during forceps-assisted vaginal delivery.

There is no consensus on the relationship between DRA, pelvic floor disorders and pelvic girdle pain. While a substantial proportion of postpartum women exhibit both POP and DRA, studies exploring their relationship are scarce and yield conflicting results (11, 27, 32–35). In a retrospective cohort of more than 200 patients in Guangdong, China, DRA severity showed no significant association with pelvic floor dysfunction, despite increasing inter-rectus distance (27). In the present study with a larger sample, higher type I pelvic floor muscle strength (Grades II–IV) was protective against DRA compared with Grade 0, and DRA severity was inversely related to POP stage. These results were somewhat unexpected, as pregnancy and childbirth are known risk factors for both DRA and POP. Moreover, pelvic floor dysfunction is not an established risk factor or a definitive symptom of DRA, and the relationship between these conditions remains uncertain. In a cohort of more than 1,400 postpartum women in China, combined supraumbilical and periumbilical DRA was associated with increased BMI, episiotomy, and perineal laceration, indicating that appropriate episiotomy use may lower DRA risk in primiparous women (26). These findings led us to hypothesize that the rectus abdominis and pelvic floor muscles, both bearing increased intra-abdominal pressure during pregnancy and delivery, may compensate for each other: separation or weakening of one could reduce stress on the other. This may explain why over half of postpartum women have both DRA and POP, yet the severity of the two conditions is inversely correlated. Consistent with our results, a recent study reported that women with DRA have weaker abdominal muscles and more abdominal pain, but no higher prevalence of pelvic floor disorders (33). Another Chinese study also revealed that women with DRA were not more likely to have weakened pelvic floor muscles (32). Consequently, stricter weight management during pregnancy may exert a protective effect against both DRA and POP, and postpartum rehabilitation for women with these conditions may be best approached through targeted, individualized strategies (36–38).

Our study provides important insights into the epidemiology of DRA in our local population and its associated risk factors as well as the correlation between DRA grades and POP severities, benefiting from a large sample size that strengthens the robustness of our findings. Our findings highlight the need for personalized postpartum rehabilitation strategies for patients with DRA and POP. The main limitations of this study are that it included only a single follow-up time point at six weeks postpartum, and the diagnosis of DRA was based solely on the IRD without a more detailed classification of anatomical DRA subtypes. Given the relatively high prevalence of DRA in our study population, the odds ratios may have overestimated the strength of the associations between the identified risk factors and the occurrence of DRA. In addition, postpartum rehabilitative methods and their potential associations with the occurrence and severity of DRA were not evaluated (26). Future studies with longer follow-up periods and interventional components are warranted to further clarify these associations and evaluate the effectiveness of tailored rehabilitation strategies.

## 6 Conclusion

DRA is highly prevalent among Chinese postpartum women and is influenced by parity, birth weight, gestational weight gain, and maternal BMI. Vaginal delivery and moderate (Grade II–IV) strength of Type I pelvic floor muscles were found to be protective against DRA. Since the severity of DRA is negatively correlated with POP stages, a compensatory interaction between the abdominal wall and pelvic floor muscles is likely. These results underscore the need for comprehensive perinatal counseling, rational weight management during pregnancy, and the development of individualized postpartum rehabilitation strategies targeting both abdominal and pelvic floor musculature.

## Data Availability

The raw data supporting the conclusions of this article will be made available by the authors, without undue reservation.

## References

[1] Werner LA Dayan M. Diastasis recti abdominis-diagnosis, risk factors, effect on musculoskeletal function, framework for treatment and implications for the pelvic floor. Curr Womens Health Rev. (2019) 15(2):86–101. 10.2174/1573404814666180222152952

[2] Cardaillac C Vieillefosse S Oppenheimer A Joueidi Y Thubert T Deffieux X. Diastasis of the rectus abdominis muscles in postpartum: concordance of patient and clinician evaluations, prevalence, associated pelvic floor symptoms and quality of life. Eur J Obstet Gynecol Reprod Biol. (2020) 252:228–32. 10.1016/j.ejogrb.2020.06.038 32623254

[3] Cavalli M Aiolfi A Bruni PG Manfredini L Lombardo F Bonfanti MT Prevalence and risk factors for diastasis recti abdominis: a review and proposal of a new anatomical variation. Hernia. (2021) 25(4):883–90. 10.1007/s10029-021-02468-8 34363190 PMC8370915

[4] Dumas GA Reid JG Wolfe LA Griffin MP McGrath MJ. Exercise, posture, and back pain during pregnancy. Clin Biomech. (1995) 10(2):98–103. 10.1016/0268-0033(95)92046-O 11415538

[5] Sperstad JB Tennfjord MK Hilde G Ellström-Engh M Bø K. Diastasis recti abdominis during pregnancy and 12 months after childbirth: prevalence, risk factors and report of lumbopelvic pain. Br J Sports Med. (2016) 50(17):1092–6. 10.1136/bjsports-2016-096065 27324871 PMC5013086

[6] Wu L Gu Y Gu Y Wang Y Lu X Zhu C Diastasis recti abdominis in adult women based on abdominal computed tomography imaging: prevalence, risk factors and its impact on life. J Clin Nurs. (2021) 30(3–4):518–27. 10.1111/jocn.15568 33207011

[7] Wan D Qin T Guo L Zhang X Wang H Zheng Z Risk factors for pelvic organ prolapse in postpartum women: a retrospective cross-sectional study in Southwest China. Front Med. (2025) 12:1–9. 10.3389/fmed.2025.1663043 PMC12537672 41127400

[8] Tung RC Towfigh S. Diagnostic techniques for diastasis recti. Hernia. (2021) 25(4):915–9. 10.1007/s10029-021-02469-7 34313855

[9] Fan ZY J Li X Cheng F Hu H Xu J Zhang Z Expert consensus on the diagnosis and treatment of postpartum diastasis recti abdominis. J Pract Clin Med. (2023) 27(4):14. 10.7619/jcmp.20230017

[10] Lin S Lu J Wang L Zhang Y Zhu C Qian S Prevalence and risk factors of diastasis recti abdominis in the long-term postpartum: a cross-sectional study. Sci Rep. (2024) 14(1):25640. 10.1038/s41598-024-76974-x 39465305 PMC11514151

[11] Li M Wang N Wang R Liang B. Ultrasonographic evaluation of diastasis recti abdominis and its association with pelvic floor dysfunction in postpartum women: a cross-sectional study of a two-year retrospective cohort. Front Med (Lausanne). (2024) 11:1441127. 10.3389/fmed.2024.1441127 39703527 PMC11655187

[12] Shen Y Zhou X He K Cai Y Zhu Y Chen H Diastasis recti abdominis: a practical and effective width-length classification based on ultrasound measurements and its clinical validation. J Ultrasound Med. (2024) 43(9):1733–44. 10.1002/jum.16508 38864261

[13] Bracale U Stabilini C Cavallaro G Pecchini F Sarno G Agresta F The Italian national consensus conference on the diagnosis and treatment of Rectus abdominis diastasis in post-gravidic women. Hernia. (2025) 29(1):213. 10.1007/s10029-025-03403-x 40576747

[14] Tian P Liu DM Wang C Gu Y Du GQ Tian JW. An ultrasound observation study on the levator hiatus with or without diastasis recti abdominis in postpartum women. Int Urogynecol J. (2021) 32(7):1839–46. 10.1007/s00192-021-04783-1 33864477 PMC8295084

[15] Hernández-Granados P Henriksen NA Berrevoet F Cuccurullo D López-Cano M Nienhuijs S European Hernia society guidelines on management of rectus diastasis. Br J Surg. (2021) 108(10):1189–91. 10.1093/bjs/znab128 34595502 PMC10364860

[16] Gynecology and the American Urogynecologic Society. Pelvic organ prolapse: aCOG practice bulletin summary, number 214. Obstet Gynecol. (2019) 134(5):1124–7. 10.1097/AOG.0000000000003520 31651830

[17] Lang LZ J Song Y Zhang X Wang J Ma Q Tong X Chinese Guidelines for the diagnosis and treatment of pelvic organ prolapse (2020 edition). Chin JObstet Gynecol. (2020) 55(5):300–6. 10.3760/cma.j.cn112141-20200106-00016

[18] Swift SE Barber MD. Pelvic organ prolapse: defining the disease. Female Pelvic Med Reconstr Surg. (2010) 16(4):201–3. 10.1097/SPV.0b013e3181f0bf1d 22453340

[19] Madhu C Swift S Moloney-Geany S Drake MJ. How to use the pelvic organ prolapse quantification (POP-Q) system? Neurourol Urodyn. (2018) 37(S6):S39–43. 10.1002/nau.23740 30614056

[20] Haylen BT de Ridder D Freeman RM Swift SE Berghmans B Lee J An international urogynecological association (IUGA)/international continence society (ICS) joint report on the terminology for female pelvic floor dysfunction. Int Urogynecol J. (2010) 21(1):5–26. 10.1007/s00192-009-0976-9 19937315

[21] Haylen BT Maher CF Barber MD Camargo S Dandolu V Digesu A An international urogynecological association (IUGA)/international continence society (ICS) joint report on the terminology for female pelvic organ prolapse (POP). Int Urogynecol J. (2016) 27(4):655–84. 10.1007/s00192-016-3003-y 26984443

[22] Bland DR Earle BB Vitolins MZ Burke G. Use of the Pelvic Organ Prolapse staging system of the International Continence Society, American Urogynecologic Society, and Society of Gynecologic Surgeons in perimenopausal women. Am J Obstet Gynecol. (1999) 181(6):1324–7; discussion 7–8. 10.1016/S0002-9378(99)70371-6 10601907

[23] Bump RC Mattiasson A Bø K Brubaker LP DeLancey JO Klarskov P The standardization of terminology of female pelvic organ prolapse and pelvic floor dysfunction. Am J Obstet Gynecol. (1996) 175(1):10–7. 10.1016/S0002-9378(96)70243-0 8694033

[24] Skaug KL Engh ME Bø K. Pelvic floor muscle training in female functional fitness exercisers: an assessor-blinded randomised controlled trial. Br J Sports Med. (2024) 58(9):486–93. 10.1136/bjsports-2023-107365 38413133 PMC11103308

[25] Sigurdardottir T Steingrimsdottir T Geirsson RT Halldorsson TI Aspelund T Bø K. Can postpartum pelvic floor muscle training reduce urinary and anal incontinence?: an assessor-blinded randomized controlled trial. Am J Obstet Gynecol. (2020) 222(3):247.e1–e8. 10.1016/j.ajog.2019.09.011 31526791

[26] Guo J Liu L Hua M Han D Tang X Wen J Analysis of diastasis recti abdominis phenotypes and related delivery factors at 42 days postpartum. Ann Med. (2025) 57(1):2523556. 10.1080/07853890.2025.2523556 40569089 PMC12203690

[27] Fei H Liu Y Li M He J Liu L Li J The relationship of severity in diastasis recti abdominis and pelvic floor dysfunction: a retrospective cohort study. BMC Womens Health. (2021) 21(1):68. 10.1186/s12905-021-01194-8 33588826 PMC7885475

[28] Liu X Wang Q Chen Y Luo J Wan Y. Factors associated with stress urinary incontinence and diastasis of Rectus abdominis in women at 6–8 weeks postpartum. Urogynecology (Phila). (2023) 29(10):844–50. 10.1097/SPV.0000000000001353 37093577 PMC10521785

[29] Edmondson SJ Ross DA. The postpartum abdomen: psychology, surgery and quality of life. Hernia. (2021) 25(4):939–50. 10.1007/s10029-021-02470-0 34309770

[30] Bixo L Sandblom G Österberg J Stackelberg O Bewö K Olsson A. Association between inter-recti distance and impaired abdominal core function in post-partum women with diastasis recti abdominis. J Abdom Wall Surg. (2022) 1:10909. 10.3389/jaws.2022.10909 38314149 PMC10831648

[31] Turan V Colluoglu C Turkyilmaz E Korucuoglu U. Prevalence of diastasis recti abdominis in the population of young multiparous adults in Turkey. Ginekol Pol. (2011) 82(11):817–21. 22384613

[32] Wang Q Yu X Chen G Sun X Wang J. Does diastasis recti abdominis weaken pelvic floor function? A cross-sectional study. Int Urogynecol J. (2020) 31(2):277–83. 10.1007/s00192-019-04005-9 31197430

[33] Gluppe S Ellström Engh M Kari B. Women with diastasis recti abdominis might have weaker abdominal muscles and more abdominal pain, but no higher prevalence of pelvic floor disorders, low back and pelvic girdle pain than women without diastasis recti abdominis. Physiotherapy. (2021) 111:57–65. 10.1016/j.physio.2021.01.008 33691943

[34] Braga A Caccia G Nasi I Ruggeri G Di Dedda MC Lamberti G Diastasis recti abdominis after childbirth: is it a predictor of stress urinary incontinence? J Gynecol Obstet Hum Reprod. (2019) 49(10):101657. 10.1016/j.jogoh.2019.101657 31783196

[35] Harada BS De Bortolli TT Carnaz L De Conti MHS Hijaz A Driusso P Diastasis recti abdominis and pelvic floor dysfunction in peri- and postmenopausal women: a cross-sectional study. Physiother Theory Pract. (2022) 38(10):1538–44. 10.1080/09593985.2020.1849476 33283590

[36] Chen B Zhao X Hu Y. Rehabilitations for maternal diastasis recti abdominis: an update on therapeutic directions. Heliyon. (2023) 9(10):e20956. 10.1016/j.heliyon.2023.e20956 37867827 PMC10589864

[37] Skoura A Billis E Papanikolaou DT Xergia S Tsarbou C Tsekoura M Diastasis recti abdominis rehabilitation in the postpartum period: a scoping review of current clinical practice. Int Urogynecol J. (2024) 35(3):491–520. 10.1007/s00192-024-05727-1 38340172 PMC11023973

[38] Beamish NF Davenport MH Ali MU Gervais MJ Sjwed TN Bains G Impact of postpartum exercise on pelvic floor disorders and diastasis recti abdominis: a systematic review and meta-analysis. Br J Sports Med. (2025) 59(8):562–75. 10.1136/bjsports-2024-108619 39694630 PMC12013572

